# Type 2 autoimmune hepatitis: Genetic susceptibility

**DOI:** 10.3389/fimmu.2022.1025343

**Published:** 2022-09-29

**Authors:** Pascal Lapierre, Fernando Alvarez

**Affiliations:** ^1^ Laboratoire d’hépatologie cellulaire, Centre de recherche du Centre hospitalier de l’Université de Montréal (CRCHUM), Montréal, QC, Canada; ^2^ Département de médecine, Université de Montréal, Montréal, QC, Canada; ^3^ Service de gastroentérologie, hépatologie et nutrition, Centre Hospitalier Universitaire (CHU) Sainte-Justine, Montréal, QC, Canada; ^4^ Département de Pédiatrie, Université de Montréal, Montréal, QC, Canada

**Keywords:** liver, autoimmunity, genetic, HLA, MHC

## Abstract

Two types of autoimmune hepatitis (AIH) are recognized; AIH-1 is characterized by the presence of anti-nuclear and/or anti-smooth muscle autoantibodies, while AIH-2 is associated with the presence of anti-Liver kidney microsome and/or anti-Liver Cytosol antibodies. The autoantigens targeted by AIH-2 autoantibodies are the cytochrome P450 2D6 and Formiminotransferase-cyclodeaminase for anti-LKM1 and anti-LC1 respectively. Both autoantigens are expressed in hepatocytes at higher levels than in any other cell type. Therefore, compared to AIH-1, the autoantigens targeted in AIH-2 are predominantly tissue-specific. Distinct clinical features are specific to AIH-2 compared to AIH-1, including diagnosis in younger patients (mean age 6.6 years), onset as fulminant hepatitis in very young patients (3 years of age or less), higher frequency in children than in adults and is frequently associated with extrahepatic T cell-mediated autoimmune diseases. AIH-2 is also often diagnosed in patients with primary immunodeficiency. AIH-2 is associated with specific HLA class II susceptibility alleles; DQB1*0201 is considered the main determinant of susceptibility while DRB1*07/DRB1*03 is associated with the type of autoantibody present. HLA DQB1*0201 is in strong linkage disequilibrium with both HLA DRB1*03 and DRB1*07. Interestingly, as in humans, MHC and non-MHC genes strongly influence the development of the disease in an animal model of AIH-2. Altogether, these findings suggest that AIH-2 incidence is likely dependent on specific genetic susceptibility factors combined with distinct environmental triggers.

## Introduction

Autoimmune hepatitis (AIH) is a chronic inflammatory liver disease with a fluctuating course, that progresses to cirrhosis and liver failure if not adequately treated. AIH shows a non-Mendelian inheritance; therefore, a single genetic locus cannot be associated with the development of the disease. However, it is believed that one or several genes, acting alone or in concert, can influence the risk of developing AIH.

Pediatric AIH has an annual incidence of 0.23 per 100000 children, and in North America, AIH-1 is 5 to 6 times more frequently diagnosed than AIH-2 (1). AIH etiology is unknown, however, it has been proposed that environmental triggers, like drugs or viruses, could be responsible for immune activation, possibly through molecular mimicry or by modification of potential autoantigens. Epitopes shared between viruses and autoantigens could be processed and presented to immune system cells in the context of specific HLAs, mainly class II molecules. Interestingly, some viruses, such as the Hepatitis A virus, can induce protracted hepatitis in patients expressing HLA class II DRB1*1301, responsible for susceptibility to the development of AIH in Argentine and Venezuelan populations (2, 3). In patients expressing predisposing HLA class II, an acute infection could trigger an immune response against liver antigens. This has led to the hypothesis that a specific susceptibility HLA class II could initiate an autoimmune response while susceptibility genes outside the HLA locus could assure the perpetuation of it, thus allowing the development of an autoimmune disease (4).

Importantly, HLA class II alleles of protection have also been identified in AIH and other autoimmune diseases. Those HLA class II would present specific epitopes preferentially stimulating regulatory T cells i.e., MHC molecules present self-epitopes that specifically activate Tregs (5).

## Autoimmune hepatitis

Autoimmune hepatitis is a disease of unknown pathogenesis where the infiltration of the liver by self-reactive lymphocytes leads to the progressive destruction of the hepatic parenchyma (6). In absence of treatment, AIH progresses to cirrhosis with a median survival time of 3.3 years (7). Clinical observations and the study of AIH etiology have led researchers to hypothesize that this disease, like the majority of autoimmune diseases, is multifactorial.

AIH is classified into two types according to the type of autoantibodies present (8). AIH-1 is defined by the presence of *anti-smooth muscle antibodies* (SMA) and/or *anti-nuclear antibodies* (ANA). AIH-2 is characterized by the presence of autoantibodies directed against cytochrome P450 2D6 (LKM1 for *anti-liver-kidney microsome type 1 antibody*) and/or against formiminotransferase-cyclodeaminase (FTCD) (LC1 for *liver-cytosol type 1 antibody*).

Type 1 and type 2 AIH show similar features such as the form of presentation, clinical signs of liver disease, presence of other autoimmune diseases in patients or first-degree relatives, an increase of immunoglobulin G in serum, female predominance, histological findings, and a favorable response to immunosuppressive drugs. Nevertheless, in a careful analysis of both types, some significant differences have been uncovered.

AIH-2 is more frequent in pediatric than in adult patients, a peak of incidence is found well before puberty, in contrast, patients with AIH-1 are usually older. In children, the mean age for onset of AIH-2 is 6.6 years and for AIH-1 10.6 years (6). The median age at presentation for AIH-1 is 12 years with an interquartile range of 11 to 14 years compared to 10 years for AIH-2 with an interquartile range of 4.5 to 13 years (1). Cases of AIH-2 presenting as an acute liver failure have a mean age of around 2 to 3 years, but of 13 to 14 years for patients with AIH-1. Female incidence is 9:1 for AIH-2 and 3:1 for AIH-1, thus AIH-2 is largely prevalent in girls (4). Extra-hepatic autoimmune diseases in AIH-2 patients are almost exclusively associated with T cell-mediated diseases; this is not the case for AIH-1 (6) (**Table 1**). In addition, AIH-2 is more frequently diagnosed than AIH-1 in patients with inherited immune deficiencies (9).

**Table 1:** Extrahepatic autoimmune diseases and Primary Immune deficiencies most frequently found in children with type 1 and 2 autoimmune hepatitis.

Type 1 AIH	Type 2 AIH
**T cell-dependent extrahepatic autoimmune diseases**	
Ulcerative colitis	Autoimmune enteropathy
Crohn’s disease	Thyroiditis^a^
Vasculitis	Vitiligo
Arthritis	Alopecia
Thrombocytopenia	Diabetes^a^
Fibrosing alveolitis	
Hemolytic anemia	
Glomerulonephritis	
**Primary Immune Deficiencies**
APECED
Autoimmune lymphoproliferative syndrome
Immunodysregulation polyendocrinopathy enteropathy x-linked (IPEX) syndrome
STAT1 deficiencies
Chronic mucocutaneous candidiasis

AIH, autoimmune hepatitis; APECED, autoimmune polyendocrinopathy-candidiasis-ectodermal dystrophy syndrome.

a These autoimmune diseases are also found in patients with type 1 AIH.

### Genes of the major histocompatibility complex and autoimmune hepatitis

The strongest genetic association with AIH was found with genes of the major histocompatibility complex (MHC). This 3.6Mb region of chromosome 6 contains nearly 260 genes involved in the immune response such as MHC class I and II genes, genes responsible for antigenic presentation, complement genes, and several cytokines.

Several susceptibility alleles for AIH have been identified (**Table 2**). In North America and Europe, MHC alleles HLA-A1-B8, HLA-DRB1*0301 (DR3), and HLA-DRB1*0401 (DR4) have been found in association with AIH (10, 11). In a linkage disequilibrium study conducted by our team in families of pediatric patients with AIH-1 and 2, we found that compared to their unaffected siblings, HLA-DRB1*0301 (DR3) and DRB1*1301 (DR13) were preferentially transmitted to patients with AIH-1, while HLA-DQB1*0201 was preferentially transmitted to children with AIH-2, compared to the randomly expected frequency or unaffected siblings (12). In a separate study, HLA-DR13 was suggested to be a risk factor in absence of HLA-DR3 or HLA-DR4 alleles (13). However, the size of the study population precluded the authors from reaching statistically significant conclusions (13).

**Table 2:** Susceptibility alleles present in patients with type 1 and 2 AIH.

Genes	Population	AIH	References
**MHC genes**			
HLA-A1-B8	North America, Europe	Type 1	Manns et al., Gastroenterology, 1994; Doherty et al., Hepatology, 1994, Ma et al., Hepatology, 2021
HLA-DRB1*0401	North America, Europe, Netherlands	Type 1	Manns et al., Gastroenterology, 1994; Doherty et al., Hepatology, 1994, De Boer et al. Gastroenterology, 2014
HLA-DRB1*0404	Mexico	Type 1	Vasquez-Garcia et al., J hepatol, 1998
HLA-DRB1*0405	Argentina, Japan	Type 1	Pando M, Larriba et al. Hepatology, 1999; Seki et al., Gastroenterology, 1992, Yoshizawa et al. J Hepatol, 2005
HLA-DRB1*1301	North America, Europe, Brazil, Argentina	Type 1	Djilali-Saiah et al., J Hepatol, 2004; Fainboim et al., Hum Immunol, 1994
HLA-B	China	Type 1	Liu et al. Hepatology, 2022
HLA-DRB1*0301	North America, Great Britain, Spain, Argentina, Netherlands	Type 1 and 2	Manns et al., Gastroenterology, 1994; Doherty et al., Hepatology, 1994; Czaja et al., Am J Gastroenterol, 1999, De Boer et al. Gastroenterology, 2014, Ma et al., Hepatology, 2021
HLA-DRB1-07	Germany, Brazil, Great Britain	Type 2	Jurado et al. J hepatol, 2002; Bittencourt et al., Am J Gastroenterol, 1999, Ma et al., Hepatology, 2021.
HLA-DRB3*01	Brazil		Czaja et al., J Hepatol, 2002; Jurado et al., J hepatol, 1997
HLA-DQB1*0201	North America, Europe	Type 2	Djilali-Saiah et al., J hepatol, 2004
HLA-DQB1*0603	North America, Europe	Type 2	Djilali-Saiah et al., J hepatol, 2004
**Non-MHC genes**			
IgA	Europe	Type 1	De la Concha et al., J Immunol, 2002; Vorechovski et al., Am J Hum Genet, 1999
TNFa*2	North America, Great Britain	Type 1	Cookson et al., Hepatology, 1999; Czaja et al. Gastroenterology, 1999
CTLA4	North America, Europe	Type 1	Agarwal et al. Hepatology, 2000
Fas	Japan, North America	Type 1	Hiraide et al., Am J Gastroenterol, 2005
CD28-CTLA4-ICOS	China	Type 1	Liu et al. Hepatology, 2022
SYNPR	China	Type 1	Liu et al. Hepatology, 2022
Vitamin D receptor	Germany	Type 1 and 2	Vogel et al., Hepatology,2002
C4A	Europe, North America	Type 1 and 2	Vergani et al., Lancet, 1985; Scully et al., Gastroenterology, 1993

Other HLA alleles have been described in association with AIH in other populations. HLA-DRB1*0404 is predominant in Mexican adult patients with AIH (14) while HLA-DRB1*0405 has been associated with Argentine and Japanese AIH patients (15, 16). In Brazil, HLA-DRB1*1301 and DRB3*01 have been found in association with AIH (17). In AIH-2, an association with HLA-DRB1*07 was found in German, Brazilian and British patients, while the HLA-DRB1*03 allele was identified as a risk factor in Spanish patient (18–20). Recently, Ma *et al.* described a unique genetic profile in juvenile patients of European descent for AIH-1 (DRB1*03), AIH-2 (DRB1*07), and autoimmune sclerosing cholangitis (DRB1*13) in addition to HLA-B*08, HLA-DRB1*03, and A1-B8-DR3 haplotype that predisposed to all three forms of juvenile autoimmune liver disease (21). Interestingly, homozygosity for DRB1*03 or DRB1*13 was associated with fibrosis at disease presentation while possession of DRB1*03, DRB1*13, and DRB1*07 alleles was associated with a more severe disease for all three forms of juvenile autoimmune liver disease (21). This diversity of susceptibility alleles found in these different ethnic groups for AIH could be explained by the “common motifs” hypothesis that proposes that several alleles of Class II HLA could code for similar motifs (22).

These associations of class II HLA alleles and AIH susceptibility may be directly related to disease pathogenesis. For example, it was found that in 94% of patients with AIH-1, susceptibility alleles encoded for the LLEQKR or LLEQRR motifs at positions 67 to 72 of the HLA class II molecule (11, 23). Interestingly, HLA-DB1*1501, which is associated with a lower risk of developing AIH-1, encodes for ILEQAR at these same positions (11, 23). The substitution at position 71 of lysine with arginine or alanine which alters both the polarity and charge of the amino acid, could cause a change in the orientation and/or binding of peptides within the MHC class II molecule. These changes could influence autoantigens’ presentation to T cells and thus modify the development of the disease.

Studying the relationship between HLA alleles and humoral response in AIH-2, our group described the strong influence that class II alleles could have on AIH patients’ autoimmune B cell responses (24). HLA-DRB1*03 was strongly associated with AIH-2 patients with circulating anti-LKM1 and anti-LC1 autoantibodies, while the HLA-DRB1*07 allele was predominant in patients with AIH-2 for which anti-LKM1 were the only serological markers present (24). In addition, patients with the HLA-DRB1*07 allele have anti-LKM1 autoantibodies against a smaller repertoire of autoepitopes compared to patients with the HLA-DRB1*03 allele (21).

HLA class II DQ2 in AIH-2 is in strong linkage disequilibrium with HLA DRB1*03 and DRB1*07 and associated with circulating LKM1 autoantibodies (18). An extremely strong association was also found between LKM1 positivity and HLA class II DR7 in patients chronically infected with HCV (18). In individuals with AIH-2 and HCV-, 7 and 9 out of 15 expressed DR7 or DR3 respectively. Additionally, among the 33 HCV+ patients with LKM1 autoantibodies studied, 28 expressed DR7 in a heterozygous or homozygous form (18). This suggests that DR7 is strongly associated with susceptibility to the development of LKM1 autoantibodies (18). Interestingly, it has already been proposed that the production of LKM1 in HCV-positive patients could be due to a molecular mimicry mechanism (25). The development of specific type 2 autoantibodies based on HLA class II alleles suggests that different environmental triggers associated with specific HLA class II susceptibility alleles could be responsible for the development of AIH-2. Interestingly, HLA class II susceptibility alleles for AIH-1 vary significantly in different regions of the world while those for AIH-2 are very similar. For example, HLA-DR7 is carried by almost 70% of patients with AIH-2 in Brazil, the other 30% expressing DRB1*03 (26).

In an interesting study, the T-cell reactivity against CYP2D6 was analyzed in relation to the patient’s HLA class II alleles to characterize their association with disease activity, cytokine production profile, and AIH clinical course (27). Analysis showed that stimulation with CYP2D6 favored a Th1 response, with the CYP2D6 peptide aa 305-324 inducing the highest levels of interferon production. This peptide is recognized by HLA DR7 and non-DR7 individuals and can be considered the T-Cell’s dominant epitope. Other epitopes, mainly recognized by HLA DR7 patients were between aa 73-124, 177-2112, and 217-260 (27). It can be concluded from these studies that an extensive overlap exists between the B and the T cell immune response, both controlled by the presentation within a particular HLA context (24, 27).

In a Genome-Wide Association Study (GWAS) of 649 adult patients with AIH-1, a significant association was found between AIH and SH2B3 and CARD10 variants in the 6p21 region of the major histocompatibility complex (28). Other genes involved in the activation of T cells, by regulating cytokines signaling or inducing cell maturation and proliferation have also been associated, however, without reaching statistical levels of significance.

Recently, in another Genome-Wide Association study of 1622 AIH-1 patients, Li *et al.* confirmed the previous association of AIH with HLA, in this case, SNP RS6932730 located in the intronic region of the HLA-B gene, but also found associations with two novel loci, CD28-CTLA4-ICOS and SYNPR (29). Interestingly, CD28-CTLA4-ICOS is a co-stimulatory receptor gene cluster located on chromosome 2q33 that encodes both the positive (CD28 and ICOS) and negative (CTLA-4) T-cell regulators (29). Unfortunately, similar Genome-Wide Association studies have not been carried out in patients with AIH-2 since their numbers are much lower (28).

### Non-MHC susceptibility genes

A long list of susceptibility genes for autoimmune diseases in humans has been identified, including genes related to lymphocyte activation and intracellular signaling, the major histocompatibility complex, cytokines, and cytokine receptors, innate immunity, microbial recognition, transcription factors, and several other pathways or mechanisms (30).

Other HLA genes have been found in association with susceptibility to AIH, such as the IgA and complement factor 4A (C4a) genes (**Table 2**) (31). IgA deficiency is frequent in patients with AIH and is genetically related to the MHC locus, specifically to HLA susceptibility alleles HLA-DR1 and HLA-DR7 (32, 33). In addition, low levels of C4a are found in 69% of children with AIH (34). This deficiency may be related to the pathogenesis of the disease: indeed, several deletions in the C4a gene have been described in patients developing the disease at a young age (35). However, it is difficult to isolate the effect of this complement gene on AIH, as this gene is in strong linkage disequilibrium with the HLA A1-B8-DR3-DQ2 susceptibility haplotype (36).

Despite their odds ratios for AIH susceptibility being significantly lower than those for HLA alleles, several genes outside the MHC locus have been linked to AIH. These genes encode proteins that can regulate innate and/or adaptive immune responses, as is particularly the case in Graves’ disease (37), multiple sclerosis (38), or celiac disease (39) where polymorphisms of the CTLA-4 gene have been found in adult and pediatric patients with AIH-1 (40, 41). CTLA-4 is a negative regulator of immune responses therefore, loss of this molecule could lead to a complex immune dysregulation syndrome, affecting several organs, including inflammatory infiltration of the liver (42). Linkage disequilibrium for this gene has also been found in affected children compared to unaffected siblings (41) Indeed, there is an increased transmission of alleles (AT)_8_ and (A) of exon 1 of the CTLA-4 gene from heterozygous parents to their child with AIH-1 (87.5% and 83.5%, respectively) compared to unaffected children (50.0% for both alleles) (41). In contrast, no difference in the transmission of these alleles was found for patients with AIH-2 and their unaffected siblings (41).

A polymorphism in the promoter region of the FAS gene at position -670 has also been found in association with the development and progression of AIH, leading to an aggressive disease with the early development of cirrhosis (43, 44).

An association between vitamin D receptor gene polymorphisms and the development of primary biliary cholangitis and AIH, two autoimmune liver diseases, has also been found (45). The vitamin D receptor is thought to have a role in several functions of the immune system such as activation of macrophages and monocytes, specific inhibition of the effector functions of CD4+ Th1 cells, and inhibition of dendritic cell differentiation (46–48). Therefore, vitamin D receptor polymorphisms could alter the immunological response to an autoantigen and potentially influence the development of an autoimmune disease. In addition, tumor necrosis factor-alpha (TNF-α) gene polymorphisms can also confer susceptibility to AIH and influence its course. Substitution of G to A at the -308 position could influence gene transcription resulting in increased constitutive and induced circulating levels (49, 50). AIH patients with this polymorphism are susceptible to early disease development, are less likely to go into remission, and are more likely to develop cirrhosis (50).

Mutations in the *autoimmune regulator* gene (AIRE), which are responsible for the development of the Autoimmune Polyendocrinopathy syndrome (APECED), can also lead to the development of AIH in 10% to 20% of cases (38, 51). The AIRE gene is a transcription factor that is central in the thymic negative selection of self-reactive T cells. Thus, mutations that impair its function can lead to multiple autoimmune manifestations by increasing the number of self-reactive T cells. Autoantibodies in those patients are usually against CYP1A2, and less frequently against CYP2D6, the autoantigen recognized by LKM1 autoantibodies (52). AIH can be an early and severe complication in these patients. An apparent association between AIRE mutations and HLA DRB1*0301-DQB1*0201 and LKM1 autoantibodies against CYP1A2 was found in individuals developing an AIH (53). A case report has also shown LKM1 autoantibodies in APECED patients with AIRE mutations and AIH that respond to immunosuppression (54).

Heterozygous AIRE gene mutations have also been found in a few patients with AIH, one with AIH-2 and another with AIH-1 (55). However, studies of known mutations in the AIRE gene in patients with autoimmune liver diseases have shown that mutations in this gene probably do not play a significant role in the pathogenesis of AIH (37, 55).

## Animal models and genetic susceptibility to autoimmune hepatitis

Animal models of AIH can be useful to study the influence of genetic background or specific mutations on the pathogenesis of AIH. An animal model of AIH-2 was developed by our group using xenoimmunization with human CYP2D6 and FTCD proteins of C57BL/6 mice (56). These mice show circulating anti-LKM1 and anti-LC1 autoantibodies, increased serum alanine aminotransferase (ALT) levels, and significant lymphocyte infiltration of the liver (56). In addition, this AIH-2 experimental model shows the same female preponderance as seen in human AIH; xenoimmunized female animals developed severe AIH while xenoimmunization of male mice resulted in minimal liver inflammation (57). Interestingly, this was associated with the development of increased levels of regulatory T cells in males compared to females (57).

Using this non-transgenic mouse model, the susceptibility to develop AIH-2 of three mouse strains (C57BL/6, 129/Sv, and BALB/c) was compared. 129S/v mice share the same class I and II MHC genes as C57BL/6 but have different non-MHC genes while BALB/c mice have different MHC and non-MHC genes. Following xenoimmunization, C57BL/6 mice developed AIH while 129s/v mice showed sparse liver lobular infiltrate and slightly elevated ALT levels while BALB/c mice showed no signs of liver inflammation (56). This experiment showed that both MHC and non-MHC genes can influence the development of experimental AIH-2 and suggests that a class II MHC haplotype (H-2^b^ in this case) is permissive but not sufficient for the development of AIH in this model (56).

An animal model of Autoimmune Polyendocrinopathy type 1 was also produced by truncating exon 2 of the *AIRE* gene. A quarter of those mice developed an AIH of intensity dependent on the *AIRE* mutation and the mouse genetic background (58). This study also suggests that genetic susceptibility to AIH is rarely linked with a single genetic locus and that a combination of susceptibility alleles is involved in the development of AIH.

A humanized mouse model expressing the DR3 HLA allele was studied after injection of the DNA plasmid coding for CYP2D6/FTCD to induce the development of AIH-2 (59). Immunized mice showed a sustained elevation of aminotransferases, LKM1/LC1 autoantibodies production, chronic inflammation, and fibrosis in the liver (59). The authors also observed an enhanced Th1 response and decreased levels of liver infiltrating Treg cells (59). These experiments using the AIH-2 animal model confirm the relevance of specific class II HLA alleles in the development of AIH and could allow the study of specific environmental triggers.

The role of Programmed cell death 1 (PD-1) on AIH susceptibility, an immunoreceptor of the CD28/cytotoxic T-lymphocyte-associated antigen 4 (CTLA-4) family that provides negative co-stimulation, has also been explored (60). PD-1^-/-^ mice do not develop spontaneous AIH despite the lack of PD-1-mediated peripheral tolerance (60). However, when a neonatal thymectomy was also performed on these mice, mice developed fatal hepatitis (60). This suggests that in addition to defects in peripheral tolerance, an impaired central tolerance and the presence of circulating autoreactive T cells are required to induce an AIH (60).

Other mouse models have also shown links between AIH susceptibility and several other genes involved in immune tolerance including the TAM subfamily of receptor tyrosine kinases (Tyro3, Axl, and Mer) (61) and TRAF6, an E3 ubiquitin-protein ligase, that influences T cell tolerance through regulation of medullary thymic epithelial cell development (62).

## Conclusion

As with most autoimmune diseases, the main genetic associations in AIH involve genes of the major histocompatibility complex and in the case of type 1 and 2 AIH specifically, HLA class II genes and the HLA-DR locus. AIH is also linked with non-HLA genes, but their odds ratios for AIH susceptibility are far lower than those for HLA alleles. AIH susceptibility has been associated with SNPs in several genes including CTLA-4, TNF-α, vitamin D receptor, and AIRE. Genetic risk factors for AIH have been made with genes involved in central and peripheral immunological tolerance that regulate the proliferation and fate of autoreactive B and T cells, cytokine production, and inflammatory and immune responses in general. Animal models have been useful to pinpoint specific genes or groups of genes however, work remains to be done to elucidate the link between genetic predisposition, the break of immunological tolerance, and the development of AIH.

## Author contributions

Both authors were responsible for the writing and revision of the article. All authors contributed to the article and approved the submitted version.

## Funding

This review was funded by an Autoimmune Hepatitis Pinnacle Research Award in Liver Disease from the American Association for the Study of Liver Diseases (AASLD) to PL.

## Conflict of interest

The authors declare that the research was conducted in the absence of any commercial or financial relationships that could be construed as a potential conflict of interest.

## Publisher’s note

All claims expressed in this article are solely those of the authors and do not necessarily represent those of their affiliated organizations, or those of the publisher, the editors and the reviewers. Any product that may be evaluated in this article, or claim that may be made by its manufacturer, is not guaranteed or endorsed by the publisher.
